# Potential for microbial H_2_ and metal transformations associated with novel bacteria and archaea in deep terrestrial subsurface sediments

**DOI:** 10.1038/ismej.2017.39

**Published:** 2017-03-28

**Authors:** Alex W Hernsdorf, Yuki Amano, Kazuya Miyakawa, Kotaro Ise, Yohey Suzuki, Karthik Anantharaman, Alexander Probst, David Burstein, Brian C Thomas, Jillian F Banfield

**Affiliations:** 1 Department of Plant and Microbial Biology, University of California, Berkeley, CA, USA; 2 Nuclear Fuel Cycle Engineering Laboratories, Japan Atomic Energy Agency, Tokai, Ibaraki, Japan; 3 Horonobe Underground Research Center, Japan Atomic Energy Agency, Horonobe, Hokkaido, Japan; 4 Graduate School of Science, The University of Tokyo, Tokyo, Japan; 5 Department of Earth and Planetary Sciences, Berkeley, CA, USA; 6 Department of Environmental Science, Policy, and Management, Berkeley, CA, USA

## Abstract

Geological sequestration in deep underground repositories is the prevailing proposed route for radioactive waste disposal. After the disposal of radioactive waste in the subsurface, H_2_ may be produced by corrosion of steel and, ultimately, radionuclides will be exposed to the surrounding environment. To evaluate the potential for microbial activities to impact disposal systems, we explored the microbial community structure and metabolic functions of a sediment-hosted ecosystem at the Horonobe Underground Research Laboratory, Hokkaido, Japan. Overall, we found that the ecosystem hosted organisms from diverse lineages, including many from the phyla that lack isolated representatives. The majority of organisms can metabolize H_2_, often via oxidative [NiFe] hydrogenases or electron-bifurcating [FeFe] hydrogenases that enable ferredoxin-based pathways, including the ion motive Rnf complex. Many organisms implicated in H_2_ metabolism are also predicted to catalyze carbon, nitrogen, iron and sulfur transformations. Notably, iron-based metabolism is predicted in a novel lineage of Actinobacteria and in a putative methane-oxidizing ANME-2d archaeon. We infer an ecological model that links microorganisms to sediment-derived resources and predict potential impacts of microbial activity on H_2_ consumption and retardation of radionuclide migration.

## Introduction

Nuclear energy is deemed to have the potential to offset greenhouse gas emissions from oil and gas power plants and combat climate change, but the wastes generated by this technology may remain highly radioactive for over 100 000 years. Currently, disposal of long-lived radioactive wastes in deep, isolated geological repositories is the only credible long-term solution for human safety (Alexander and McKinley, 2011; Cherry *et al.*, 2014). However, uncertainties exist around the impacts of these repositories on host rock and groundwater, especially over the geologic time frames required for containment. Conversely, the combination of site-specific geology, hydrology, geochemistry and microbiology are all predicted to provide containment after the eventual breakdown of engineered barrier systems (Harrison *et al.*, 2011; Suzuki *et al.*, 2016).

Microorganisms, ubiquitous components of deep terrestrial ecosystems, thrive on resources such as buried organic carbon, mineral-associated electron donors and acceptors, and biologically or abiotically formed hydrogen and methane (Edwards *et al.*, 2012; Pedersen, 2013). In turn, microbial communities can influence the mineralogical and geochemical features of their environments (Lovley and Chapelle, 1995; Pedersen, 1997; Edwards *et al.*, 2012). Importantly, microbial activities can also impact radioactive waste disposal in the subsurface. For example, microbial respiration can form and maintain reducing conditions following the closure of the disposal facility (Puigdomeneck *et al.*, 2001; Amano, *et al.*, 2012a). Additionally, H_2_ produced by corrosion of metals associated with repository materials may stimulate H_2_-oxidizing bacteria and archaea whose activities may reduce the risks associated with H_2_ accumulation (Libert *et al.*, 2011; Vinsot *et al.*, 2014; Bagnoud *et al.*, 2016). Some likely H_2_ oxidizers include sulfate reducers that generate sulfide that could induce rapid corrosion of containment materials (Rajala *et al.*, 2015), and metal reducers that may reductively immobilize radionuclides (Mohapatra *et al.*, 2010; Williams *et al.*, 2011; Williamson *et al.*, 2013). Furthermore, biofilm development within the pore spaces or fractures of host rocks could affect radionuclide transport and gas diffusion (Pedersen, 2005).

These microbially catalyzed processes also depend on geologic features. For instance, fracture networks in granitic rocks could allow rapid diffusion of gaseous H_2_ away from a repository, whereas the low permeability of sedimentary rocks could impede H_2_ diffusion (Cherry *et al.*, 2014). Research at the granite-hosted Äspö hardrock laboratory has revealed active microbial H_2_ oxidation and sulfate reduction *in situ* as well as uranium immobilization by biofilms (Krawczyk-Bärsch *et al.*, 2012; Pedersen, 2013). Wu *et al.* (2015) recently applied genome-resolved metagenomics to this site to develop a metabolic model of the microbial community, and suggested a higher dependency on organic carbon than previously considered for hardrock environments. Less is known about how microbial communities in sedimentary rock formations may affect repository safety, although research on the Opalinus Clay and Boom Clay formations suggests that the members and complexity of sediment-hosted communities vary immensely by site (Wouters *et al.*, 2013; Bagnoud *et al.*, 2015, 2016).

Understanding microbially mediated processes in sedimentary rocks holds broad significance for global biogeochemical cycling. Deep terrestrial sediments are a vast reservoir of buried organic carbon that is subject to microbial transformation (Breuker *et al.*, 2011; Mcmahon and Parnell, 2014), often resulting in the formation of carbon dioxide or methane (Pedersen, 2000). Underground research laboratories (URLs) such as Horonobe URL provide the opportunity to study deep subsurface systems. The Horonobe URL has been constructed by the Japan Atomic Energy Agency to conduct basic geoscientific research and evaluate the feasibility and safety of geological disposal in deep sedimentary environments. The URL is situated above Neogene rocks consisting of marine sandstones, mudstones and shales (Hama *et al.*, 2007). Previous microbiological studies at Horonobe URL found a diversity of Proteobacteria and methanogens inhabiting the groundwater, as determined by sequencing of 16S ribosomal RNA (rRNA) gene libraries (Shimizu *et al.*, 2006; Kato *et al.*, 2009). Laboratory studies using rock samples collected from 200 m below ground surface and inoculated with *Pseudomonas denitrificans* supported the development of biofilm communities at *in situ* groundwater conditions (Harrison *et al.*, 2011; Sakurai and Yoshikawa, 2012). However, comprehensive information about the microbial community structure and metabolic potential of Horonobe groundwaters has not been obtained.

Here, we conducted deep metagenomic sequencing of nine groundwater samples collected from the URL at depths of 140–250 m below the surface. We reconstructed genomes from the sequence data and explored the nature of the subsurface biosphere by incorporating community metabolic potential into a conceptual ecological model. Finally, we used this information to predict how the deep subsurface sediment-associated community could impact radioactive waste repository function in the long term.

## Materials and methods

### Site description

**Figure 1:**
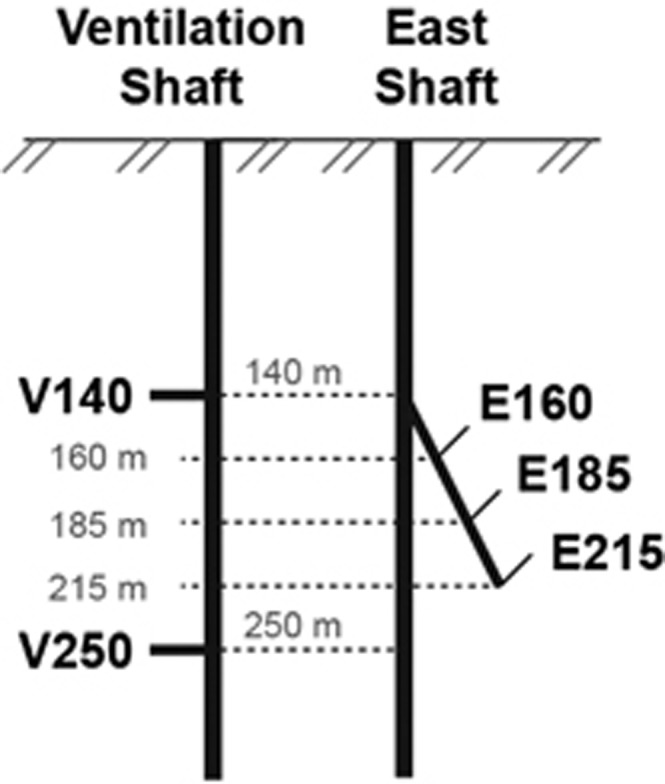
Sampling locations within the Horonobe URL. Abbreviations used in the text follow the format [V/E][Depth][Year], where V/E refers to Ventilation/East Shafts.

Groundwater and headspace gas samples were collected from five depths 140–250 m below ground surface at the Horonobe URL (45°02'43"N, 141°51'34"E; Figure 1). At the sampled depths, the rock was composed primarily of soft diatomaceous mudstones, including opal-A and trace amounts of quartz, feldspar, clay minerals, pyrite, calcite and siderite (Hiraga and Ishii, 2007; Ishii *et al.*, 2007; Tachi *et al.*, 2011). The groundwater is 14–18 °C in temperature, brackish and of circumneutral pH. The porewater geochemistry of this site has been determined previously (Sasamoto *et al.*, 2011, 2014; Amano *et al.*, 2012b), and is summarized in Supplementary Table S1. Within the Horonobe URL, three main shafts provide access to subsurface galleries, where boreholes have been drilled into the rock faces. Boreholes sampled during this investigation include 07V140M03 and 09V250M02 drilled from the Ventilation shaft, and three depths within 08E140C01 drilled from the East shaft. All groundwater samples were obtained using a multipacker system developed at the Horonobe URL (Nanjo *et al.*, 2012).To eliminate the influence of drilling and the installation of tools for hydrochemical monitoring (for example, sampling tubes, and casing pipes), groundwaters were drained at least five times the section volume, before sampling for geochemical and microbial studies. The groundwater chemistry has been monitored since 2008, starting immediately after the drilling of these boreholes. Based on the results of geochemical analyses, the compositions of the groundwaters are very stable (Sasamoto *et al.*, 2011, 2014; Amano *et al.*, 2012b). The quality of each groundwater sample was checked by measurement of the concentration of sodium naphtionate, which is an indication of drilling fluid contamination. The concentrations were below the detection limit in all samples.

### Headspace gas sampling

Groundwater samples were collected between June 2012 and June 2013, and analyzed for gas composition using a water displacement procedure under atmospheric pressure. The composition of the gas samples (H_2_, N_2_, O_2_, CH_4_, CO_2_ and C_2_H_6_) was determined by gas chromatography. H_2_, N_2_, O_2_ and CH_4_ were analyzed using a micro-gas chromatograph (GC) (Micro-GC CP-2002; Varian Chrompack International, Agilent Technologies, Middelburg, The Netherlands) packed with a Molecular Sieve-5A and micro-thermal conductivity detector. Oxygen was used as the carrier gas for H_2_ analysis, and Ar was used for N_2_, O_2_ and CH_4_ analyses, as described previously (Miyakawa *et al.*, 2010). CO_2_ and C_2_H_6_ were analyzed using a micro-GC (490 Micro-GC; Agilent Technologies) equipped with a Pora-PLOTQ column with He as the carrier gas and micro-thermal conductivity detector. The column temperature and pressure of the 490 Micro-GC were maintained at 80 °C and 190 kPa, respectively. Analytical precision was determined by a 95.4% (two sigma) confidence interval of standard gas or air analyses (*n*=60); H_2_ is 1.8%, O_2_ is 7.9%, N_2_ is 9.0%, CH_4_ is 8.3%, CO_2_ is 2.4% and C_2_H_6_ is 2.8%.

### Biological sampling, DNA extraction and sequencing

Two groundwater samples were collected for metagenomics during 2014 from Ventilation shaft boreholes, one each from boreholes 07V140M03 and 09V250M02. An additional seven samples were collected for metagenomics at two times during 2013 and 2014 from borehole 08E140C01. All samples were collected by passage of groundwater through 0.22 μm membrane filters (type GVWP; Merck Millipore, Darmstadt, Germany). One sample (E215m-2014_f, obtained during 2014 from 215 m depth in borehole 08E140C01) was collected by passage of the 0.22 μm filtrate through a 10 000 nominal molecular weight limit filter (type PLGC; Merck Millipore). Other samples were also collected by this method, but insufficient DNA was recovered for metagenomic analyses. The volume of groundwater samples used for filtration was between 0.9 and 38 L, depending on the cell densities in each groundwater sample. DNA was extracted from the biomass retained on the filters using the Extrap Soil DNA Kit Plus ver. 2 (Nippon Steel and Sumikin Eco-Tech Corporation, Tsukuba, Japan). Genomic DNA libraries were generated using TruSeq Nano DNA Sample Prep Kit (Illumina, San Diego, CA, USA) according to the manufacturer’s instructions. Library quality was examined using an Agilent 2100 bioanalyzer (Agilent Technologies) and paired-end 150-bp reads with a 550 bp insert size were sequenced by Hokkaido System Science Co., Ltd (Hokkaido, Japan). using an Illumina HiSeq2000 (San Diego, CA, USA). Sample volumes, DNA concentrations and sequencing information are presented in Supplementary Table S2. All sequencing data were deposited in the NCBI database (BioProject ID: PRJNA321556).

### *De novo* genomic assembly, read mapping and annotation

Raw shotgun sequencing reads were trimmed using the adaptive read trimmer, Sickle (Joshi and Fass, 2011), with default settings. All samples were assembled *de novo* using IDBA-UD with the following parameters: —mink 40, —maxk 100, —step 20 and —pre_correction (Peng *et al.*, 2012). Trimmed shotgun sequencing reads from each sample were mapped to all scaffolds >1000 bp, using Bowtie2 with default parameters (Langmead *et al.*, 2009). Further details of the curation process are described in the Supplementary Materials. For all scaffolds over 1000 bp, open reading frames were predicted with Prodigal using the meta setting (Hyatt *et al.*, 2012). Functional annotations for all open reading frames were predicted by BLAST (Altschul *et al.*, 1990) searches against the Uniref100 (Suzek *et al.*, 2007), Uniprot (Magrane and UniProt Consortium, 2011) and KEGG (Kanehisa *et al.*, 2012) databases as described previously (Raveh-Sadka *et al.* 2015). tRNA sequences were predicted using tRNAscan-SE (Schattner *et al.*, 2005), and 16S rRNA sequences were identified using the cmsearch program from the Infernal package (Nawrocki *et al.*, 2009).

### Binning and conserved gene analysis

Assembled scaffolds >1000 bp were binned by a combination of phylogenetic profiles, read coverage and nucleotide content (that is, GC proportion and tetranucleotide signatures) within the binning interface of ggKbase (http://ggkbase.berkeley.edu/) and by using emergent self-organizing maps constructed separately from tetranucleotide frequencies and coverage of the scaffolds across the nine different assemblies (Dick *et al.*, 2009; Sharon *et al.*, 2013). Details of the binning procedure are provided in the Supplementary Materials. Resulting bins were evaluated for accuracy and completeness in ggKbase using a set of conserved single-copy phylogenetic marker genes as described previously (Raveh-Sadka *et al.*, 2015; Probst *et al.*, 2016). De-replication of bins was determined using pairwise identity comparisons with Nucmer (Kurtz *et al.*, 2004) via scripts described previously (Probst *et al.*, 2016).

### Sequence alignment and phylogeny

Alignment of 16S rRNA genes with reference sequences from the Silva SSU database v.123 was performed with SSU-ALIGN (Nawrocki, 2009) using default parameters, followed by refinement and removal of insertion sequences as described previously (Brown *et al.*, 2015). A set of 16 ribosomal proteins used for phylogenetic placement were identified by profile hidden Markov model (HMM) searches seeded with HMM profiles from the Phylosift conserved phylogenetic marker genes (Darling *et al.*, 2014). Amino-acid sequences for ribosomal protein were added to a database of ribosomal protein sequences clustered at the genus level (Hug *et al.*, 2016) and aligned using MAFFT (Katoh *et al.*, 2002). The alignments were then manually trimmed in Geneious v.8 (BioMatters Ltd., San Francisco, CA, USA) to remove poorly aligned positions at the N and C termini and columns composed of over 90% gaps. The alignments were then concatenated, and sequences with fewer than 50% of all aligned positions were removed before tree construction. Trees were built using RAxML v.8.1.24 (Stamatakis, 2006) on the CIPRES web server (Miller *et al.*, 2010), with the PROTGAMMALG model of evolution and number of bootstraps replicates determined automatically by the extended majority rule.

### Taxonomic assignment of bins

All bins were identified to appropriate taxonomic levels in the following order: (i) identification of 16S rRNA genes against the Silva SSU database v.123 (Pruesse *et al.*, 2007); (ii) grouping of concatenated ribosomal protein sequences against the aforementioned database (Hug *et al.*, 2016); (iii) taxonomic clustering of annotated genes by protein BLAST searches against Uniref100, Uniprot and KEGG databases. In cases where taxonomic assignments disagreed among the three methods, bins were assigned to the most specific taxonomic level for which the three methods agreed. Bins in which a majority of the genes had no taxonomic affiliation or that matched viruses were annotated as potential extrachromosomal elements (plasmids and phages).

### Estimated coverage of community

We identified a set of unique marker genes by clustering the ribosomal protein S3 (RpS3) assembled across the sampling points. RpS3 was used in place of 16S rRNA genes because of the higher reliability of assembling this gene in metagenomic data sets (Castelle *et al.*, 2013), and its utility as a conserved phylogenetic marker.

### Functional gene analysis

Metabolic coding potential of individual bins was explored by reciprocal protein BLAST searches against the aforementioned protein databases and HMM searches against protein families downloaded from FunGene (Fish *et al.*, 2013), TIGRFAM v.14.0 (Haft *et al.*, 2003) and Pfam v.28.0 (Finn *et al.*, 2014), as well as custom-built profile HMMs for several target genes (Eddy, 2011). Conserved marker genes were used to indicate the potential for autotrophy, carbon degradation and biogeochemical cycles as listed in Supplementary Table S3.

Hydrogenase cofactors were identified as [NiFe], [FeFe] or [Fe] using HMM profiles and aligned with references sequences from (Greening *et al.*, 2015) for further classification. Group A3 [FeFe] hydrogenases were differentiated from Group A1 by searching for downstream genes required for Group A3 function: hydB—a diaphorase ortholog containing multiple [4Fe-4S] clusters; hydC—a small protein containing a [2Fe-2S] cluster (Buckel and Thauer, 2013). In contrast, Group A1 [FeFe] hydrogenases are monomeric or have non-catalytic subunits lacking the redox active [4Fe-4S] and [2Fe-2S] clusters found in Group A3 (Greening *et al.*, 2015).

Multiheme cytochromes (MHCs) are predicted to be involved in iron transformations, and can be identified by the conserved heme-binding motif, CXXCH. To estimate the validity of using MHCs as a signal for iron reduction, we downloaded a representative proteome of each prokaryotic genera in the RefSeq database as of 30 October 2015 (Tatusova *et al.*, 2014). Each protein in the reference proteome (and metagenome) was scanned for CXXCH motifs, and labeled as an MHC if it contained 10 or more motifs. Reference genomes containing 4 or more MHCs were investigated to determine if they were from organisms previously identified as capable of dissimilatory iron reduction (Supplementary Table S4).

## Results and discussion

### Metagenomic reconstruction of groundwater communities

**Figure 2:**
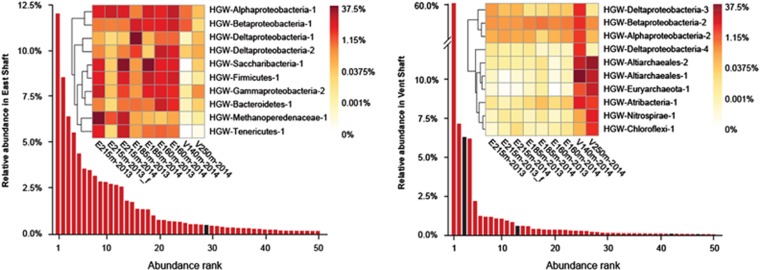
Bars represent the rank abundance curve of 50 most abundant unique *rps3* genes detected in Horonobe assemblies. Red bars indicate scaffolds that were confidently assigned to a genome bin, and black bars indicate scaffolds that remained unbinned. The heights of bars indicate scaffold coverage as a fraction of East shaft (left) and Ventilation shaft (right) communities. Heatmaps show per-sample genome abundance of 10 most abundant genomes from East shaft (left) and Ventilation shaft (right).

Groundwater samples for metagenomics were obtained from three boreholes at the URL from a depth range of between 140 and 250 m in two consecutive years. Illumina sequencing of the extracted DNA generated a total of 123.6 Gbp of raw paired-end sequence (Supplementary Table S2). We filtered and *de novo* assembled the reads and then binned the assembled sequence using a combination of methods (GC, coverage, taxonomic affiliation, cross-sample abundance and tetranucleotide frequency) to obtain a total of 550 genome bins. De-replication of these bins based on pairwise identity resulted in 228 distinct bins, of which 161 were considered to be high-quality draft genomes (>70% complete; <10% of potential contamination; Supplementary Table S5). The genomes retrieved represent a vast majority of the abundant taxa in the groundwater. Specifically, these genomes represent 49 of the 50 most abundant organisms identified in the East Shaft samples and 46 of the 50 most abundant organisms identified in the Ventilation Shaft samples (Figure 2). Of the organisms missed by our metagenomic approach, 82% are predicted to comprise <0.1% of the total community, based on sequence abundance information.

**Figure 3:**
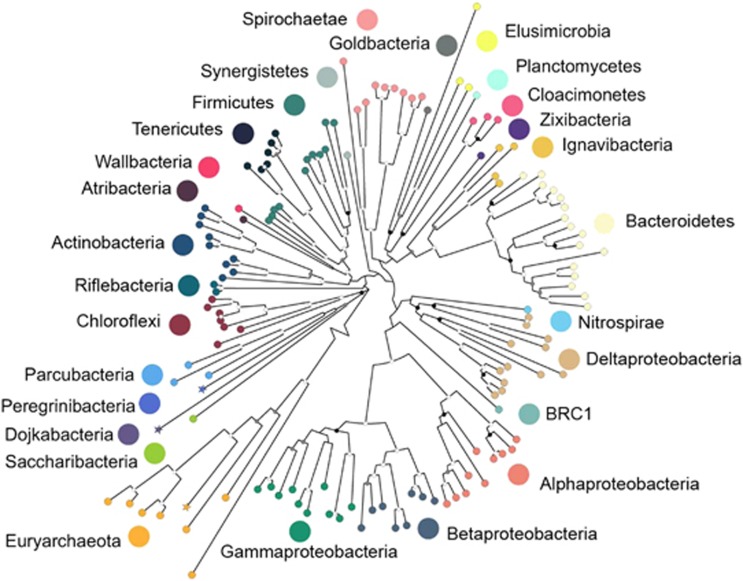
Phylogenetic tree of 130 high-quality genomes constructed from concatenated sequences of 16 ribosomal proteins. Sequences were excluded from the tree if they contained fewer than 450 aligned positions. Support for internal nodes was constructed from 100 bootstrap replicates (white⩾50%, gray⩾75%, black⩾95% confidence, no shading⩽50%). Stars represent essentially complete genomes.

Taxonomic assignment of the genomes determined that 15 are from Archaea, 195 are from Bacteria and 19 are from phage or other mobile elements (Figure 3 and Supplementary Figures S1–S3). The communities are highly diverse, with genomic representation of 29 phyla, 13 of which currently lack cultivated representatives and were missed in prior 16S rRNA gene surveys of this site (Shimizu *et al.*, 2006; Kato *et al.*, 2009). Even the genomes from organisms affiliated with relatively well-studied phyla were phylogenetically novel, as over 120 genome bins could not be taxonomically placed beyond the family level (Supplementary Table S5).

### Extensive potential for hydrogen metabolism

**Figure 4:**
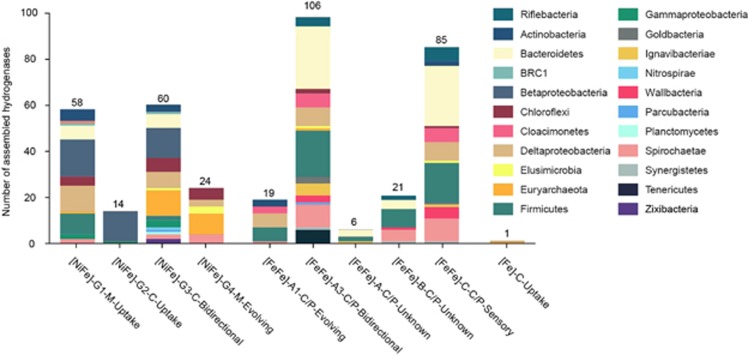
Number of hydrogenases detected in the Horonobe genomes, by phylum and class, in the case of Proteobacteria. Hydrogenase type is annotated as [Cofactor]-Group-Localization (M=membrane-bound, P=periplasmic, C=cytoplasmic)-Function.

A notable feature of the genomes was extensive potential for H_2_ metabolism, supporting prior indications that H_2_ is an important energy currency in the subsurface (Brazelton *et al.*, 2012). A total of 394 hydrogenases were detected in 119 of 161 genomes, and across 20 phyla (Figure 4). To evaluate the functional role and directionality of these genes in H_2_ metabolism, we identified the cofactor (that is, [NiFe], [FeFe] or [Fe]) and classified each hydrogenase using HMM profiles and phylogenetic association with reference databases (Greening *et al.*, 2015).

[FeFe] hydrogenases were distinguished into three major groups (A, B and C) by sequence identity and Group A was further subdivided based on operon structure, as phylogeny alone was insufficient to distinguish the function of [FeFe] Group A hydrogenases. Surprisingly, ~80% of the [FeFe] Group A hydrogenases were identified as heterotrimeric/heterotetrameric Group A3 [FeFe] hydrogenases, which reversibly bifurcate electrons from H_2_ to ferredoxin and NAD (Greening *et al.*, 2015). Another 15% were of monomeric composition (Group A1), whereas the remaining 5% could not be reliably assigned to A1 or A3 because the genes were located adjacent to the end of the scaffold. The high occurrence of bifurcating hydrogenases found at Horonobe has not been recognized in studies at other sites, although bioinformatics studies have suggested that a significant fraction of H_2_-producing anaerobes harbor bifurcating hydrogenases (Schut and Adams, 2009; Greening *et al.*, 2015). However, a comparison of the abundance of bifurcating and monomeric [FeFe] hydrogenases in environmental systems has not been possible until recently, as genomic context is required to differentiate the function of the catalytic subunits.

**Figure 5:**
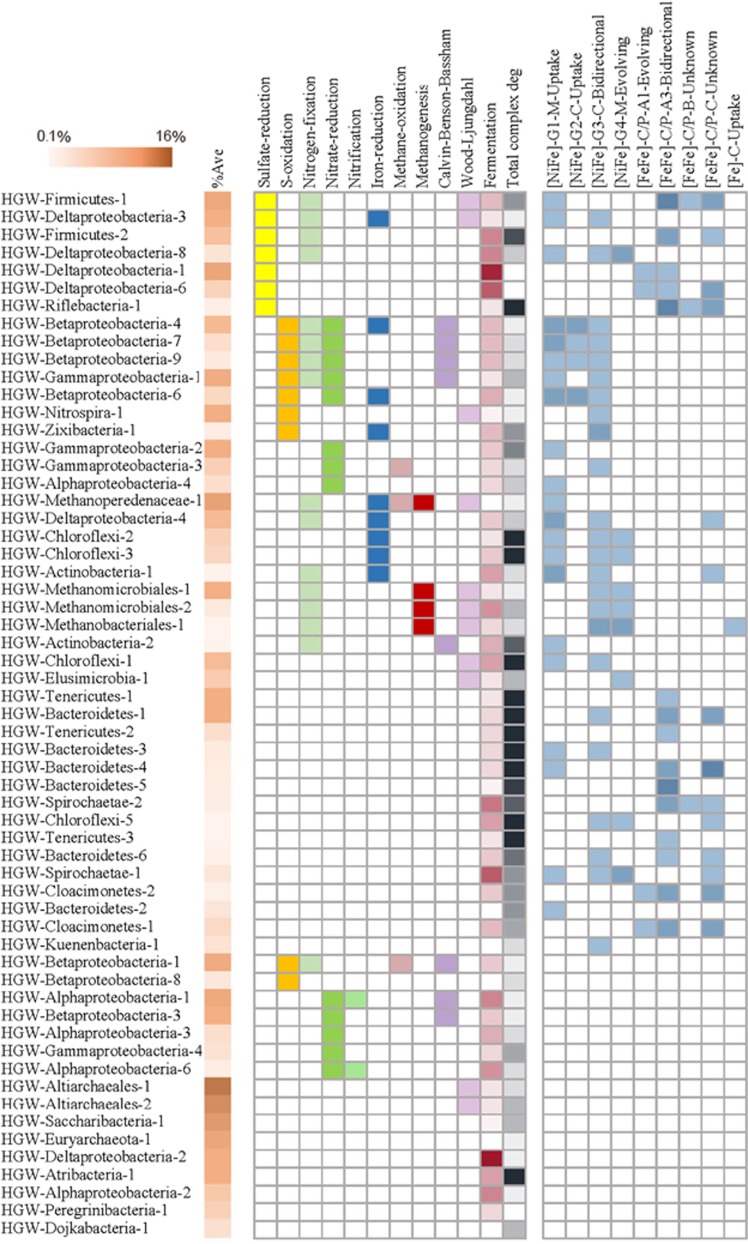
Metabolic potential to influence biogeochemical cycles of 59 genomes with an average abundance >0.1% across all samples. Differentially shaded tiles in the %Ave column represent average relative abundance of the genomes as a fraction of the complete community. Differentially shaded tiles for fermentation, complex carbon degradation and hydrogenases indicate the relative number of genes involved in such processes.

Although CH_4_ and CO_2_ comprised a vast majority (>99%) of gases obtained from all the sampling zones (Table 1), H_2_ levels were also consistently elevated across the sampling zones (11.6–36.4 p.p.m.) during a multiyear sampling effort. The high levels of H_2_ in the Horonobe subsurface make H_2_oxidation a favorable strategy for generating reduced cofactors like ferrodoxin and NAD, and has likely enriched for organisms with genomes containing bifurcating hydrogenases. In support of this, Rnf complexes were detected in over 70% of the genomes with bifurcating [FeFe] hydrogenases, which could allow the direct coupling of hydrogen oxidation to generation of a proton gradient for ATP synthase (Biegel *et al.*, 2011; Tremblay *et al.*, 2013). Additionally, the few monomeric, H_2_-evolving [FeFe] hydrogenases (Group A1) that were identified nearly always co-occurred with bifurcating hydrogenases (Figure 5 and Supplementary Figure S4). This high degree of co-occurrence suggests that organisms that evolve H_2_ (that is, those that have A1 hydrogenases) may also rely upon uptake hydrogenases (that is, A3 hydrogenases) to fuel their metabolism in times of substrate limitation. In previous metagenomic surveys of H_2_-metabolizing communities, [FeFe] hydrogenases have been frequently taken as an indication of H_2_ production (Brazelton *et al.*, 2012). Indeed, the *in situ* direction of bifurcating hydrogenases cannot be determined from sequence alone, as they could function in the reverse direction as confurcating hydrogenases (Sieber *et al.*, 2012). However, the abundance of bifurcating [FeFe] hydrogenases and co-occurrence of Group A1 with A3 hydrogenases suggests that the presence of [FeFe] hydrogenases alone does not indicate H_2_ production. Similar patterns have also been observed recently in studies of human stool metagenomes (Wolf *et al.*, 2016). These results have important implications when interpreting the results of read-based, rather than genome-based metagenomics.

[FeFe] Group B hydrogenases, whose function is currently unknown, were also identified in 17 genomes, but were found exclusively in organisms that also harbored bifurcating Group A3 hydrogenases. Group C putative sensory [FeFe] hydrogenases were likewise abundant and only identified in genome bins that also had genes for H_2_ uptake or evolution. The high incidence of Group B and C [FeFe] hydrogenases from genomes at Horonobe suggest these enzymes are highly important in environmental systems, and points to the need to further characterize this prevalent group.

A total of 155 [NiFe] hydrogenases were identified across the four major [NiFe] hydrogenase groups, most of which were found in groups commonly considered to have uptake or bidirectional functions. As with the [FeFe] hydrogenases, the genomic evidence for H_2_uptake in [NiFe] hydrogenases reflects the value of H_2_ as a source of reducing power in the deep subsurface and suggests that elevated H_2_ concentrations have enriched for hydrogenotrophs.

### Metals cycling catalyzed by novel lineages

Sediment-associated ferric iron-bearing minerals, including smectite and chlorite, and mineral coatings also provide potential resources for microbial metabolism. To evaluate the microbial potential to reduce such minerals, we searched the genomes for MHCs (operationally defined as cytochromes with 10 or more heme-binding motifs). MHCs have been identified as components of electron transferring complexes in metal-reducing bacteria like *Geobacter sulfurreducens* and *Shewanella oneidensis* (Weber *et al.*, 2006), and may be general predictors of iron reduction capacity given that organisms with many MHCs are almost exclusively iron-reducing bacteria and archaea. By scanning the RefSeq genome database for MHCs, we found that 1.2% of the unique species in the database contained 4 or more MHCs, of which 90% have been shown or predicted to be capable of dissimilatory iron reduction (Supplementary Table S5). Using these criteria on our metagenome dataset, we identified 253 MHCs from 63 genomes, and predict that 23 genomes encode the capacity for iron reduction (Figure 5 and Supplementary Figure S4).

Interestingly, the genome of the most abundant organism in the E215m zone is an ANME-2d archaeon (HGW-Methanoperedenaceae-1) that encodes 11 MHCs, four of which include 50 or more heme-binding motifs. To our knowledge, these represent the most heme-rich cytochromes sequenced to date in any organism. Although other ANME lineages have recently been hypothesized to use MHCs for direct transfer of electrons to syntrophic Deltaproteobacteria partners (McGlynn *et al.*, 2015), the abundance of HGW-Methanoperedenaceae-1 across our samples is not significantly correlated with any other organism (using an abundance threshold of 10 : 1). The HGW-Methanoperedenaceae-1 genome does not encode the ability to use nitrate or sulfate as electron acceptors, although it has a complete methanogenesis/methane-oxidation pathway. Thus, we suggest that the MHCs are used in the direct reduction of iron minerals coupled to methane oxidation. These predictions are further supported by multivariate functional analyses that validated the correlation between total Fe in groundwater and the abundance of HGW-Methanoperedenaceae-1 (Supplementary Figure S5). The inference is consistent with a very recent report linking ANME-2d archaea to iron reduction in a freshwater enrichment culture from a Dutch canal (Ettwig *et al.* 2016).

Other organisms that were enriched in the E215m zone at lower abundance included several H_2_ oxidizing iron reducers. Dissimilatory metal reduction coupled to H_2_ oxidation is often associated with Gram-negative bacteria (for example, species of *Geobacter* and *Shewanella*), and indeed, a majority of the genomes capable of metal reduction in the RefSeq database are Proteobacteria. However, Firmicutes of the Peptococcaceae family also use this metabolism (Junier *et al.*, 2010; Wrighton *et al.*, 2011). Interestingly, the four Horonobe genomes containing the most MHCs belong to Gram-positive organisms whose genomes also harbor one or more membrane-bound H_2_uptake hydrogenases [NiFe], which can liberate electrons for metals respiration (Greening *et al.*, 2015). The genome with the most MHCs is from a member of the Peptococcaceae, but surprisingly, the other three genomes were all from organisms distantly related to isolated members of the Actinobacteria. Currently isolated Actinobacteria capable of dissimilatory iron reduction are obligate acidophiles and lack MHCs in their genomes (Bridge and Johnson, 1998; Itoh *et al.*, 2011; Stackebrandt, 2014). The Horonobe clade diverges deeply from Coriobacteriobacteriales isolates, although their 16S rRNA gene sequences are closely related to those detected in iron enrichment cultures sampled from pond sediments and rice paddy soils (Supplementary Figure S6; Wang *et al.*, 2009; Lentini *et al.*, 2012). These genomes may represent an important group of neutrophilic iron-reducing organisms that has previously gone unnoticed.

### Sulfur cycling

We explored the potential for sulfur cycling by looking for key genes in pathways for sulfate reduction and oxidation of reduced sulfur compounds. In total, 20 organisms, generally representatives from the Betaproteobacteria, are predicted to conserve energy from the oxidation of reduced sulfur species, including sulfide, elemental sulfur and thiosulfate (Figure 5 and Supplementary Figure S4). An additional 15 organisms are capable of reducing sulfate; all are affiliated with the Deltaproteobacteria, Firmicutes and Riflebacteria (previously known as ACD39; Anantharaman *et al.*, 2016).

The abundance of organisms capable of sulfur cycling was not anticipated from the groundwater geochemistry, given that sulfate and sulfide concentrations in Horonobe groundwaters are consistently below 3 μm. However, analysis of porewaters extracted directly from samples of the rock matrix revealed levels of sulfate that were at least 2 orders of magnitude higher than in the groundwater (Kunimaru *et al.*, 2010). Although the high levels of sulfate detected in the porewaters were attributed to sample disturbance via oxidation of pyrite (Sasamoto *et al.*, 2011), microbially catalyzed oxidation of pyrite could liberate free sulfate, which is likely to be rapidly scavenged. The abundance of organisms capable of oxidizing and reducing sulfur species may support a cryptic sulfur cycle, where the active cycling of sulfur between solid and dissolved forms keeps concentrations low.

### Nitrogen cycling

Evidence for nitrogen cycling was investigated by searching the genomes for genes involved in nitrogen fixation, nitrate reduction, nitrification and anammox. Nitrogen fixation potential was detected in 37 genomes, which together comprise roughly one-third of the community by abundance and include four of the ten most abundant genomes from the East shaft (Figure 5 and Supplementary Figure S4).

Nitrate reduction is predicted to be a common metabolic capacity, with 72 genomes encoding at least one step in the nitrate reduction pathway. However, the pathway was frequently found to be incomplete, as only nine genomes have all the genes necessary to complete the denitrification pathway to N_2_, and 19 are capable of dissimilatory nitrite reduction to ammonia. Significant overlap exists between these two pathways, and 13 genomes are capable of reducing nitrite using either pathway.

In contrast to the number of genomes predicted to encode nitrate reduction pathways, only four genomes were predicted to encode partial nitrification pathways (each genome has a hydroxylamine dehydrogenase homolog, but lacks ammonia monooxygenase), suggesting that nitrification is not a major energy-conserving pathway in Horonobe sediments. Furthermore, hydroxylamine dehydrogenase has been shown to catalyze the reverse reaction *in vitro*, and so may be acting as a means of detoxification when nitrite levels are elevated (Kostera *et al.*, 2008). No copies of the hydrazine oxidoreductase gene were identified, and so annamox is likewise not predicted to be a capacity of any of the studied organisms.

Given that nitrate reduction capacity is frequently encoded in the studied genomes, nitrate-reducing microorganisms are likely capable of scavenging this compound at low levels. The activity of such microorganisms has resulted in depletion of nitrate, to the extent that nearly all nitrogen in both groundwater and porewater samples was present as N_2_ or ammonia. Further, the persistence of ammonia may be the consequence of a microbial biosphere in which nitrification pathways are essentially absent.

### Carbon cycling

Genes involved in carbon fixation were identified in roughly one quarter of the genome bins, representing 43 and 77% of the East shaft and Ventilation shaft communities by abundance (Figure 5 and Supplementary Figure S4). Notably, the groundwater samples collected from Ventilation shaft were dominated by an archaeon from the recently described order Altiarchaeales (Probst *et al.*, 2014). The presence of this organism would have been missed in prior 16S rRNA gene surveys of Horonobe groundwaters as commonly used primer pairs fail to amplify the 16S rRNA gene of this group (Rudolph *et al.*, 2001). Altiarchaeales were predicted to use the Wood–Ljungdahl for carbon fixation (Probst *et al.*, 2014). This pathway was the most frequently encoded strategy for carbon fixation, detected in 24 genomes mainly from Deltaproteobacteria, Firmicutes, and Archaea, including the methanogens. The abundance of autotrophs using the Wood–Ljungdahl pathway has also been found in another deep terrestrial environment (Magnabosco *et al.*, 2015), suggesting that this pathway may be an important link in the deep carbon cycle.

The methanogen genomes are all predicted to couple CO_2_ reduction to hydrogenotrophy based on the uptake hydrogenases in their genomes, and none were capable of using acetate or methanol for methanogenesis. Each methanogen genome also harbors one or more formate dehydrogenase homologs, and so may be capable of oxidizing formate produced via fermentative reactions, although the capacity for formate utilization cannot be predicted solely by the presence of formate dehydrogenase (Wood *et al.*, 2003). Regardless of the electron donor, methanogenesis is predicted to be the predominant source of methane in these depths, based on carbon and hydrogen isotope ratios of methane measured in dissolved gases (Tamamura *et al.*, 2014). This biogenic methane likely supports the growth of anaerobic methane oxidizers, such as HGW-Methanoperedenaceae-1.

The only other carbon fixation pathway identified was the Calvin cycle, as determined by the presence of RuBisCO. Interestingly, one complete (curated, closed) genome from a member of the recently described Candidate Phyla Radiation (Brown *et al.*, 2015), HGW-Dojkabacteria-1 (the second complete genome of this phylum previously referred to as WS6; Wrighton *et al.*, 2016), was found to harbor genes for both form II/III and form III-like RuBisCO that are implicated in the archaeal-type nucleotide salvage pathway, a phenomenon not reported previously. This result is surprising, given that the genome is <800 kbp in length, and suggests the importance of CO_2_ incorporation into nucleotide-derived sugars in the metabolism of this organism.

Heterotrophic lifestyles were inferred based on a lack of genes for CO_2_ fixation and presence of genes for organic carbon degradation. Based on a lack of genes for the TCA cycle, electron transport chain or other respiratory enzymes, the most abundant organism detected in the East Shaft, HGW-Saccharibacteria-1 (from the Candidate Phyla Radiation phylum previously referred to as TM7), is predicted to be a fermenter. HGW-Saccharibacteria-1 is also likely dependent on the groundwater community for survival, due to the near-complete lack of essential biosynthetic pathways (for example, for biosynthesis of amino acids, nucleotides and lipids) in its genome, a prediction that is consistent with those for other representatives of this group (Kantor *et al.*, 2013). A similar lifestyle is predicted for a bacterium affiliated with the Peregrinibacteria (a Candidate Phyla Radiation phylum) for which we reconstructed a complete (curated, closed) genome.

Complex carbon (for example, cellulose, chitin, lignin and aromatic amino acids) degradation was a common functional trait predicted for the Horonobe organisms (Supplementary Table S6). Over half the draft genomes encode genes predicted to degrade one or more forms of cellulose, hemicellulose and chitin. Cellulolytic- and chitin-degrading enzymes were particularly common in Chloroflexi, Bacteroidetes and Ignavibacteria, although genomes from several poorly characterized phyla also had extensive capacity to degrade complex carbon. In particular, a genome from a previously unrecognized phylum encodes 12 putative cellulases. We propose that the name ‘Goldbacteria’ is reserved for this phylum, should further discoveries support the inference that this is a distinct major lineage in Domain Bacteria. This suggestion is in recognition of Thomas Gold’s contributions to the study of the deep biosphere. The genome also has multiple cellobiases and other hemicellulose and endohemicellulose-debranching enzymes, making it a likely degrader of plant-derived organics. *N*-alkane monooxygenases were also detected in 12 Betaproteobacteria and Gammaproteobacteria, along with pathways for degradation of aromatic compounds, including nitrobenzene, aniline, benzoate, napthalene and toluene. Notably, five of these genomes were from organisms most closely related to the known aromatic compound-degrading organism, *Dechloromonas aromatica* (Coates *et al.*, 2001), and each maintain several pathways for breakdown of benzene via catechol cleavage by catechol 2,3-dioxygenase. However, given the lack of oxygen in the Horonobe subsurface, the organisms related to *D. aromatica* may rely on an alternative mechanism for degrading aromatic compounds, although the genes involved in this pathway have yet to be identified (Salinero *et al.*, 2009).

Genes and pathways involved in degradation of complex carbon compounds likely degrade recalcitrant organic materials trapped during sedimentation (Ishii *et al.*, 2011). In particular, the silica skeletons of diatoms buried in sediments may have locked away organics that provide carbon for microbial metabolism for millions of years (Parkes *et al.*, 2014). Additionally, the salinity profiles in the vicinity of the boreholes suggest dilution of the original seawater with freshwater (Hama *et al.*, 2007), which could bring surface-derived organics, such as humic substances, into contact with deep groundwater communities. Intermittent lack of access to such resources and the availability of dissolved inorganic carbon may also select for autotrophic organisms that continue to add to the carbon pool.

### A conceptual ecological model of the deep sedimentary subsurface

**Figure 6:**
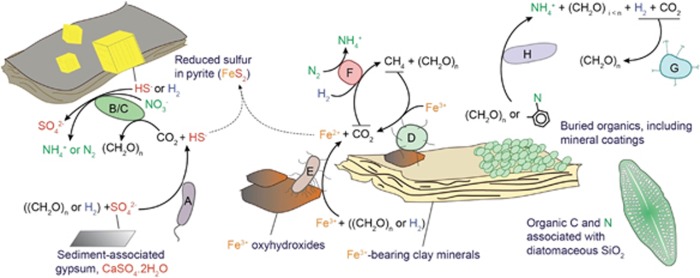
A proposed ecological model of the Horonobe subsurface. Labeled microbial populations are described in the main text.

Taken together, these genome-based metabolic predictions provide insight into the metabolic foundation for the biosphere in a deep sedimentary environment. According to the previous studies on the mineralogy of diatomaceous siliceous sedimentary rocks in the Horonobe area, the dominant minerals are quartz, feldspars, pyrite and carbonate (siderite and/or magnesite). Also present are clay minerals, including kaolinite, smectite, illite and/or chlorite (Hiraga and Ishii, 2007; Ishii *et al.*, 2007; Tachi *et al.*, 2011). Modeling of groundwater geochemistry using PhreeqC predicts that some Fe-rich clay minerals, such as smectite, nontronite and daphnite, are oversaturated, as are pyrite, Fe(OH)_3_ and goethite (Supplementary Table S7). These calculations are consistent with the observed mineralogy of rock core samples from the Horonobe site. The previously reported geological, geochemical and mineralogical information, combined with microbial results obtained here, were integrated into a conceptual model to describe the links between sediment resources and microbial ecology (Figure 6).

The abundant organic carbon buried in diatomaceous sediment provides fuel for respiratory organisms (Figures 6a and e) and fermenters (Figure 6h), which generate CO_2_, H_2_ and simple organic compounds. The buried organic materials are also likely to be an important source of nitrogen compounds, as N_2_ gas concentrations are extremely depleted in comparison with the Earth’s atmosphere. We infer that biomass-derived nitrogen is assimilated, as there is no evidence for ammonia oxidation capacity in any organism detected from any sampled site. This apparently precludes operation of a complete nitrogen cycle.

H_2_ and organics produced by fermentation can serve as electron donors to iron (Figure 6e) and sulfate (Figure 6a) reducers. Sulfate reducers depend on the availability of sulfate, which may be derived from gypsum (Figure 6a) formed, in some cases, by pyrite oxidation occurring during the construction of a repository (Figures 6b and c). However, gypsum was undersaturated in all groundwaters, based on geochemical modeling (Supplementary Table S7), and it is very rarely detected in sediments. Therefore, sulfate reduction is probably limited by low availability of sulfate. H_2_ is also capable of fueling carbon fixation (Figures 6b, c and g), which can provide a feedback loop for regeneration of organic carbon. H_2_ is also used as a substrate for methanogenesis (Figure 6f). Anaerobic methane oxidation may be coupled to iron reduction, as suggested previously (Beal *et al.*, 2009; Chang *et al.*, 2012; Ettwig *et al.*, 2016). We infer that methane-oxidation by ANME-2d archaea is coupled to reduction of ferric iron derived from sediment-associated iron-bearing minerals.

## Conclusions

This study has substantially increased genomic resolution compared with prior deep subsurface studies, with draft genomes for 80% of detected organisms. This level of genomic sampling enabled a detailed analysis of metabolic potential and pathway configurations in the sediment-associated microbial communities. The results uncovered a variety of interesting metabolic capacities, including large MHCs likely indicative of iron reduction in both Actinobacteria and methane-oxidizing archaea. We also found evidence of fermentation-based metabolisms in organisms inferred to be obligate symbionts of bacteria, and no evidence for the oxidative branch of the nitrogen cycle. Hydrogen-based metabolism, often mediated by [FeFe] bifurcating hydrogenases and Rnf complexes, is widely distributed and likely central to microbial community function.

The results reveal possible feedbacks into long-term safety of geological disposal in sedimentary environments. Elevated H_2_ levels may develop during storage of radioactive waste in some geological repositories. This gas may stimulate activity of hydrogenotrophic organisms, reducing the potential for gas accumulation in the low-diffusivity sedimentary rock environment (Vinsot *et al.*, 2014). Increased H_2_ is expected to stimulate organisms that interact directly with other biogeochemical cycles. Numerous hydrogenotrophic sulfate reducers are likely to produce sulfide. Even though dissolved concentrations of sulfur and sulfide may be low initially, construction of a geological repository involves a significant disturbance of local groundwater conditions and heat from the radioactive decay of buried wastes, and will likely drive extensive groundwater circulation. These disturbances could result in oxidation of FeS_2_ minerals and liberation of sulfate into the groundwater. Alternatively, sulfate could be supplied from distantly located sources, including oxidized zones closer to the surface. The microbial production of sulfide would accelerate the corrosion of radioactive waste containers (Rajala *et al.*, 2015). Hydrogenotrophic iron-reducing organisms may also affect the migration of radionuclides in the sedimentary rock environments (Wall and Krumholz, 2006; Junier *et al.*, 2009). The abundance of MHCs in the genomes supports the potential for enzymatic reduction of U(VI) (Marshall *et al.*, 2006) and abiotic reduction of U(VI) by biologically reduced iron (Wu *et al.*, 2010). ^99^Tc, a long-lived fission product of ^235^U, can likewise be reduced by biogenic Fe(II) and enzymatic activity of [NiFe] hydrogenases (De Luca *et al.*, 2001). Laboratory and field investigations have established that the subsurface migration of long-lived radionuclides is profoundly influenced by such enzymatic pathways (Suzuki *et al.*, 2002; Wu *et al.*, 2010; Williams *et al.*, 2011). Stimulation of metal-reducing communities could likewise retard the movement of dissolved radionuclides during the lifetime of the repository. The results presented here elucidate the potential for microbial communities to affect geochemical processes relevant for nuclear waste disposal in deep sedimentary environments and the role of hydrogen in both potentially positive and negative processes. The findings also provide insight regarding the microbiology of a deep subsurface sedimentary environment and identify potential metabolic roles for a variety of relatively little known bacteria and archaea.

## Figures and Tables

**Table 1:** Headspace gas composition of groundwater samples

	*V140m,* n=*10*	*E160m,* n=*14*	*E185m,* n=*11*	*E215m,* n=*12*	*V250m,* n=*13*
CH_4_ (%)	88.8 (±3.4)	85.5 (±8.0)	97.7 (±3.4)	99.4 (±1.8)	88.6 (±1.7)
CO_2_ (%)	10.5 (±3.0)	13.8 (±6.3)	2.8 (±2.6)	1.2 (±0.5)	9.6 (±3.3)
N_2_ (%)	0.50 (±0.54)	0.49 (±0.30)	0.46 (±0.16)	0.44 (±0.10)	0.58 (±0.34)
O_2_ (%)	0.04 (±0.03)	0.06 (±0.06)	0.05 (±0.07)	0.03 (±0.02)	0.05 (±0.04)
H_2_ (p.p.m.)	11.6 (±22.5)	33.0 (±58.7)	36.4 (±90.2)	32.8 (±46.1)	22.7 (±38.6)
C_2_H_6_ (p.p.m.)	54 (±29)	40 (±30)	39 (±30)	31 (±29)	44 (±36)

Values presented indicate the mean and standard deviation of measurements taken approximately once a month between June 2012 and June 2013.
