# Supplementary material for: Potential for microbial H2 and metal transformations associated with novel bacteria and archaea in deep terrestrial subsurface sediments
Source: ISME J. 2017 Mar 28;11(8):1915–29. doi: 10.1038/ismej.2017.39 (PMC5520028; doi:10.1038/ismej.2017.39)
Supplement: Supplementary Information [file ismej201739x1.docx]

**Assembly Curation:**

Following the read assembly and mapping protocol described in the main text, the resulting scaffolds and mappings were curated using scripts from the ra2 suite ([github.com/christophertbrown/fix_assembly_errors](https://github.com/christophertbrown/fix_assembly_errors))). Using the script “mapped.py” with the parameters -m 1 -p both, reads were filtered from SAM files if a read’s mate pair was mapped but the read was unmapped, or if a read contained more than one mismatch with the reference base. The resulting SAM files were converted to BAM files to assess coverage of each scaffold using samtools idxstats. All assemblies were then automatically curated for misassemblies using the ra2 suite.

Due to the time burden of visually scanning contigs for mapping artifacts, manual curation of the assemblies was only conducted for candidate genome bins which were of especially high quality (i.e. low contig number and high completeness). Manual curation of genomes for completeness involved establishing unique paired read coverage over the genomes by visualizing the mapping files in Geneious v8 (BioMatters Ltd) and manually modifying the contigs using the tools therein.

## Binning methods:

Assembled scaffolds >1000 bp were initially binned by phylogenetic profiles, read coverage, and GC content within the binning interface of ggKbase ([ggkbase.berkeley.edu/)](http://ggkbase.berkeley.edu/)). Tetranucleotide ESOMs were constructed for each assembly by running the script “prepare_esom_files.pl” ([github.com/CK7/esom](https://github.com/CK7/esom)) with the following settings: -k 4 -w 5000 -m 5000. Coverage-based ESOMs were constructed for each assembly by running “prepare_esom_files.pl” with the following settings: -w 5000 -m 5000 -sg <sam-files-glob>, where a SAM file glob path contains a SAM generated by mapping each read set to each assembly and filtering the mapping using *mapped.py* as above. The resulting LRN files were then normalized using the RobustZT option in Databionic ESOM Tools (Ultsch and Moerchen, 2005) and trained using the default parameters specified by Dick *et al.*, 2009 and outlined online ([github.com/tetramerFreqs/Binning](https://github.com/tetramerFreqs/Binning)). The resulting ESOM files were then loaded into Databionic ESOM Tools and overlayed with the candidate bin assignments from ggKbase using the script “class_wrapper.py” ([github.com/alexherns/biotite-scripts.git](https://github.com/alexherns/biotite-scripts.git))). The ESOMs were segregated into clusters, and written to class files that were transformed into tab-delimited scaffold-to-bin assignments using the script “cls_to_tsv.py” ([github.com/alexherns/biotite-scripts.git](https://github.com/alexherns/biotite-scripts.git))). The scaffold-to-bin assignments were finally re-evaluated in the binning interface of ggKbase.

**Conserved gene analysis:**

Determination of genome completeness was carried out in ggKbase using a set of conserved single-copy phylogenetic marker genes (51 in Bacteria and 38 in Archaea) as described previously (Raveh-Sadka *et al.*, 2015; Probst *et al.*, 2016). Identification of single-copy marker genes was based on annotations as generated by BLAST searches against the databases described in the main text. The full list of phylogenetic marker genes used is available at [github.com/alexherns/biotite-scripts/tree/master/scgs](https://github.com/alexherns/biotite-scripts/tree/master/scgs).

**HMM databases used:**

HMMs for the 16 ribosomal proteins used for phylogenetic placement are available from the Phylosift conserved phylogenetic marker genes (Darling *et al.*, 2014), and are listed at [github.com/alexherns/biotite-scripts/blob/master/rib_prots.json](https://github.com/alexherns/biotite-scripts/blob/master/rib_prots.json).

The HMM profiles used for functional gene analysis are generally available from public databases via the accession numbers listed in Supplementary Table S3. HMM profiles from Anantharaman *et al.*, 2016 will soon be made available at [github.com/banfieldlab](https://github.com/banfieldlab). HMM profiles for [FeFe] hydrogenases were generated by downloading the protein sequences from Greening *et al.*, 2015, aligning the sequences using MAFFT with default settings, and then manually trimming in Geneious v8 (BioMatters Ltd) to remove poorly aligned positions at the N- and C-termini and columns composed of over 90% gaps. The resulting alignments were then converted to HMM profiles using the “hmmbuild” command from HMMER3 (Eddy, 2011) and are available at [github.com/alexherns/biotite-scripts/tree/master/fefe_greening](https://github.com/alexherns/biotite-scripts/tree/master/fefe_greening). HMM profiles listed with KEGG entry IDs (e.g. isoamylase – K01214) were generated by David Burstein from protein sequences available in the KEGG database and will be made available in a future publication.

**Cytochrome searches:**

Heme binding domains were identified in protein sequences from the assembled metagenome and RefSeq (Tatusova *et al.*, 2014) using the script “motif_search.py” ([github.com/alexherns/biotite-scripts.git](https://github.com/alexherns/biotite-scripts.git))) with the flags: -m heme_binding -min 10 --list.

**Geochemical modeling**

The geochemical program Phreeqc (version 3.2.2, Parkhurst and Appelo, 1999) with the LLNL database was used to predict the aqueous speciation and saturation indices of mineral precipitates that could be expected when the groundwaters in the Horonobe site was at geochemical equilibrium.

**Multivariate function analyses**

In order to clarify correlations among environmental factors, principal correspondence analysis (PCA) with 25 geochemical variables was performed by R (R Core Team, 2013) for groundwater samples listed in Table 1 and Supplementary Table 1. PCA analysis revealed the geochemical similarity between V140 and E160, which is characterized by high electronic conductivity (EC) values (Fig. S?a). Deeper samples with relatively low EC values were geochemically similar except for V250 (Fig. S?a). We identified chloride, hydrogen and total Fe to be representative geochemical parameters, which were used to perform canonical correspondence analysis (CCA) with the vegan package (Oksanen *et al.* 2016) in R. To show correlation between the three variables and microbial populations, we analyzed genome-resolved taxa shown in Figure 2. Monte-Carlo Test based on 499 replicates was also performed using the ade4 package (Dray *et al.* 2015) in R to calculate a p-value. As the hypothesis that environmental factors affect the taxa distribution is statistically validated with a p-value less than 0.05, p-value of 0.03 obtained in this CCA analysis support the correlation between environmental and microbiological parameters. It is clear from Figure S?b that Methanoperedenaceae-1 and Tenericutes-1 were correlated with total Fe, whereas Chloroflexi-1, Nitrospira-1 and Euryarchaeota-1 were correlated with chloride.

**References:**

Anantharaman K, Brown CT, Hug LA, Sharon I, Castelle CJ, Probst AJ, *et al.* (2016). Metabolic handoffs shape biogeochemical cycles mediated by complex microbial communities. *Nat Commun* In Review.

Darling AE, Jospin G, Lowe E, Matsen FAI, Bik HM, Eisen JA. (2014). PhyloSift: phylogenetic analysis of genomes and metagenomes. *PeerJ* **2**: e243.

Dick GJ, Andersson AF, Baker BJ, Simmons SL, Thomas BC, Yelton AP, *et al.* (2009). Community-wide analysis of microbial genome sequence signatures. *Genome Biol* **10**: R85.

Eddy SR. (2011). Accelerated Profile HMM Searches. *PLoS Comput Biol* **7**: e1002195.

Dray, S., Dufour, A. B. & Thioulouse, J. (2015). ade 4: Analysis of Ecological Data: Exploratory and Euclidean methods in Environmental sciences. R package version 1.7-2. http://www.r-project.org/web/packages/ade4/.

Greening C, Biswas A, Carere CR, Jackson CJ, Taylor MC, Stott MB, *et al.* (2015). Genomic and metagenomic surveys of hydrogenase diversity indicate H2 is a widely-utilised energy source for microbial growth and survival. *ISME J* doi: 10.1038/ismej.2015.153.

Oksanen, J. *et al* (2016)*.* Package 'vegan'. http://www.r-project.org, <http://vegan.r-forge.r-project.org/>.

Parkhurst DL, Appelo CAJ. (1999) User’s guide to PHREEQC (Version 2) - a computer program for speciation, batch reaction, one-dimensional transport and inverse geochemical calculation. *Water-resources Investigation Report* 99–4259, USGS, Denver, Colorado, p. 312.

Probst AJ, Castelle CJ, Singh A, Brown CT, Anantharaman K, Sharon I, *et al.* (2016). Genomic resolution of a cold subsurface aquifer community provides metabolic insights for novel microbes adapted to high CO2 concentrations. *Environ Microbiol* Accepted article.

R Core Team (2013). R: A language and environment for statistical computing. R Foundation for Statistical Computing [http://www.r-project.org](http://www.r-project.org/).

Raveh-Sadka T, Thomas BC, Singh A, Firek B, Brooks B, Castelle CJ, *et al.* (2015). Gut bacteria are rarely shared by co-hospitalized premature infants, regardless of necrotizing enterocolitis development. *Elife* **4**: e05477.

Tatusova T, Ciufo S, Fedorov B, O’Neill K, Tolstoy I. (2014). RefSeq microbial genomes database: new representation and annotation strategy. *Nucleic Acids Res* **42**: D553–D559.

Ultsch A, Moerchen F. (2005). ESOM-Maps: tools for clustering, visualization, and classification with Emergent SOM.
