# Supplementary material for: Potential for microbial H2 and metal transformations associated with novel bacteria and archaea in deep terrestrial subsurface sediments
Source: ISME J. 2017 Mar 28;11(8):1915–29. doi: 10.1038/ismej.2017.39 (PMC5520028; doi:10.1038/ismej.2017.39)

Supplementary Figures

Figure S1: Concatenated ribosomal protein tree of genome bins from this study, along with reference sequences from Hug et al, 2016. Tree was generated on the CIPRES computing portal using RAxML v8.1.24 with the PROTGAMMA tree inference model, LG amino acid substitution model, and number of bootstrap replicates determined automatically by the extended majority rule.

Figure S2: 16S rRNA gene tree of archaeal sequences from this study, aligned under the archaeal 16S SSU rRNA HMM of SSU-ALIGN with reference sequences and the top 3 closest hits in a BLAST search of the SILVA SSU database v123.

Figure S3: 16S rRNA gene tree of bacterial sequences from this study, aligned under the bacterial 16S SSU rRNA HMM of SSU-ALIGN with reference sequences and the top 3 closest hits in a BLAST search of the SILVA SSU database v123.

Figure S4: Metabolic potential to influence biogeochemical cycles of all genomes of high-quality. Differentially shaded tiles in the %Ave column represent average relative abundance of the genomes as a fraction of the complete community. Differentially shaded tiles for fermentation, complex carbon degradation, and hydrogenases indicate the relative number of genes involved in such processes.

Figure S5: **a**, Principal component analysis (PCA) diagram showing the relationships among environmental factors and between environmental factors and overall groundwater properties of borehole intervals. Arrows and squares indicate the vectors of environmental factors and the plots of groundwater samples, respectively. The proportion of variations explained by each principal component is shown in parentheses along the axis. **b**, Canonical correspondence analysis (CCA) diagram showing the correlation of selected environmental factors with overall microbial communities and specific taxa. Circles and squares indicate the plots of specific taxa and overall microbial communities. The east shaft and ventilation shaft samples are highlighted in red and blue, respectively.

Figure S6: 16S rRNA gene tree of Actinobacteria sequences from this study falling within the Coriobacteriaceae clade, aligned under the bacterial 16S SSU rRNA HMM of SSU-ALIGN with reference sequences and the top 3 closest hits in a BLAST search of the SILVA SSU database v123. Sequences with accession numbers indicate environmental 16S sequences that have yet to be isolated or genome-sequenced.

Hernsdorf et al.,
Figure S1

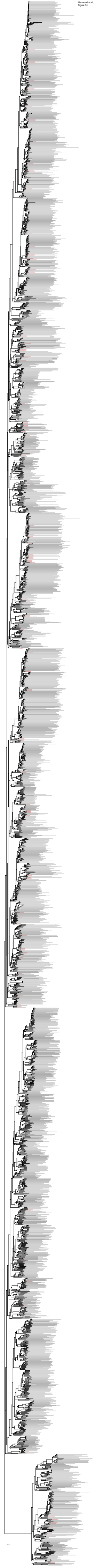

Hernsdorf et al.,
Figure S2

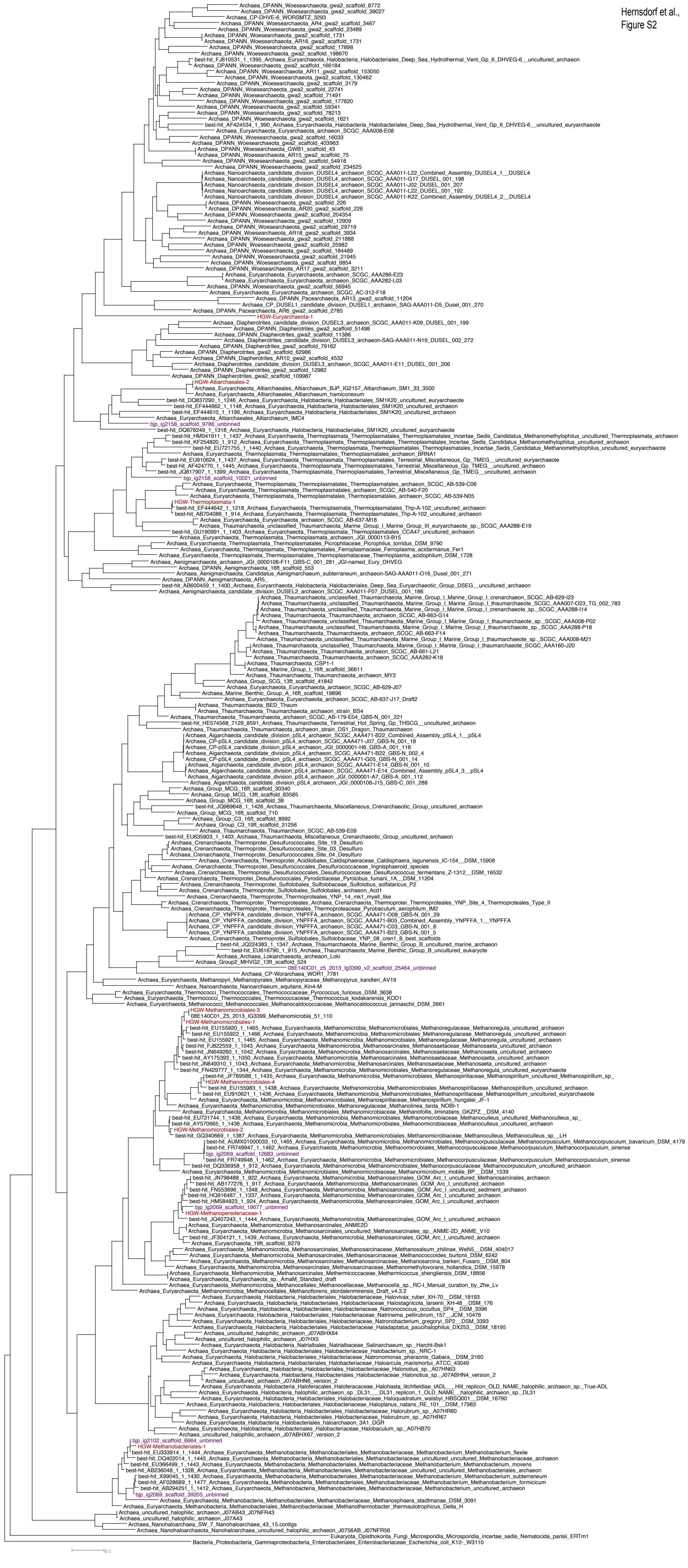

Hernsdorf et al.,
Figure S3

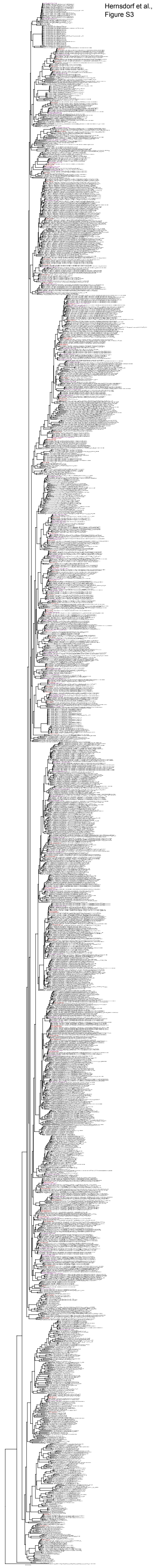

Hernsdorf et al.,

Figure S4

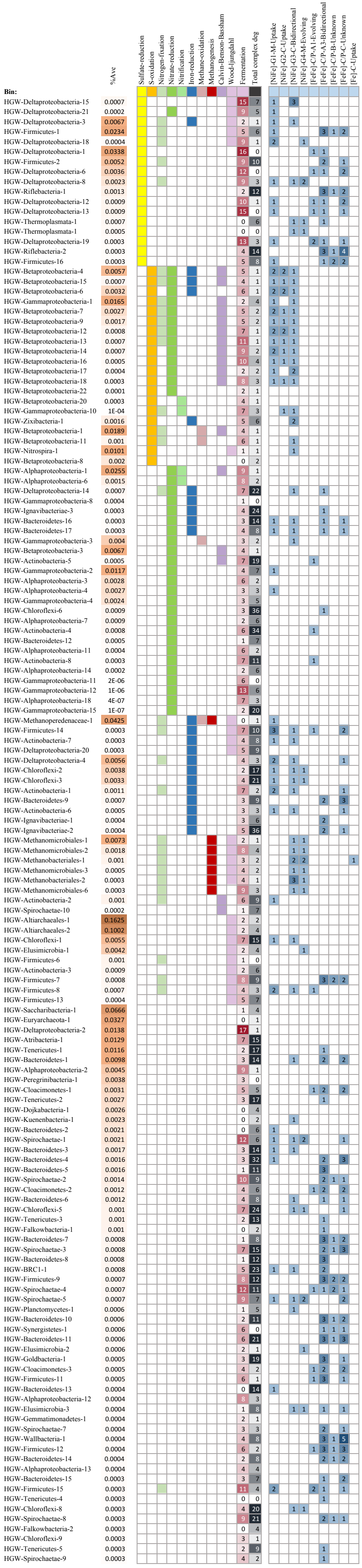

Hernsdorf et al.,
Figure S5

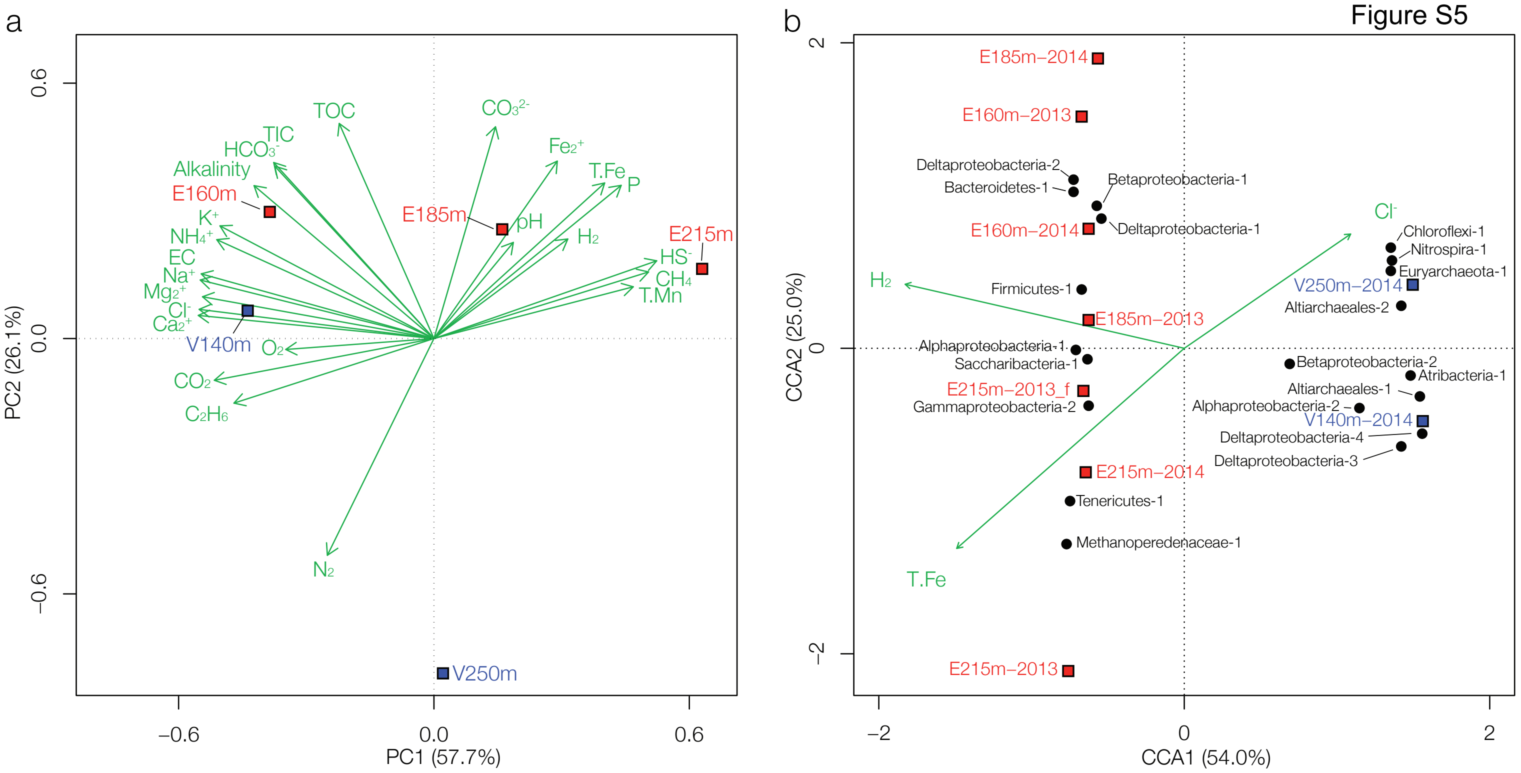

Hernsdorf et al.,
Figure S6

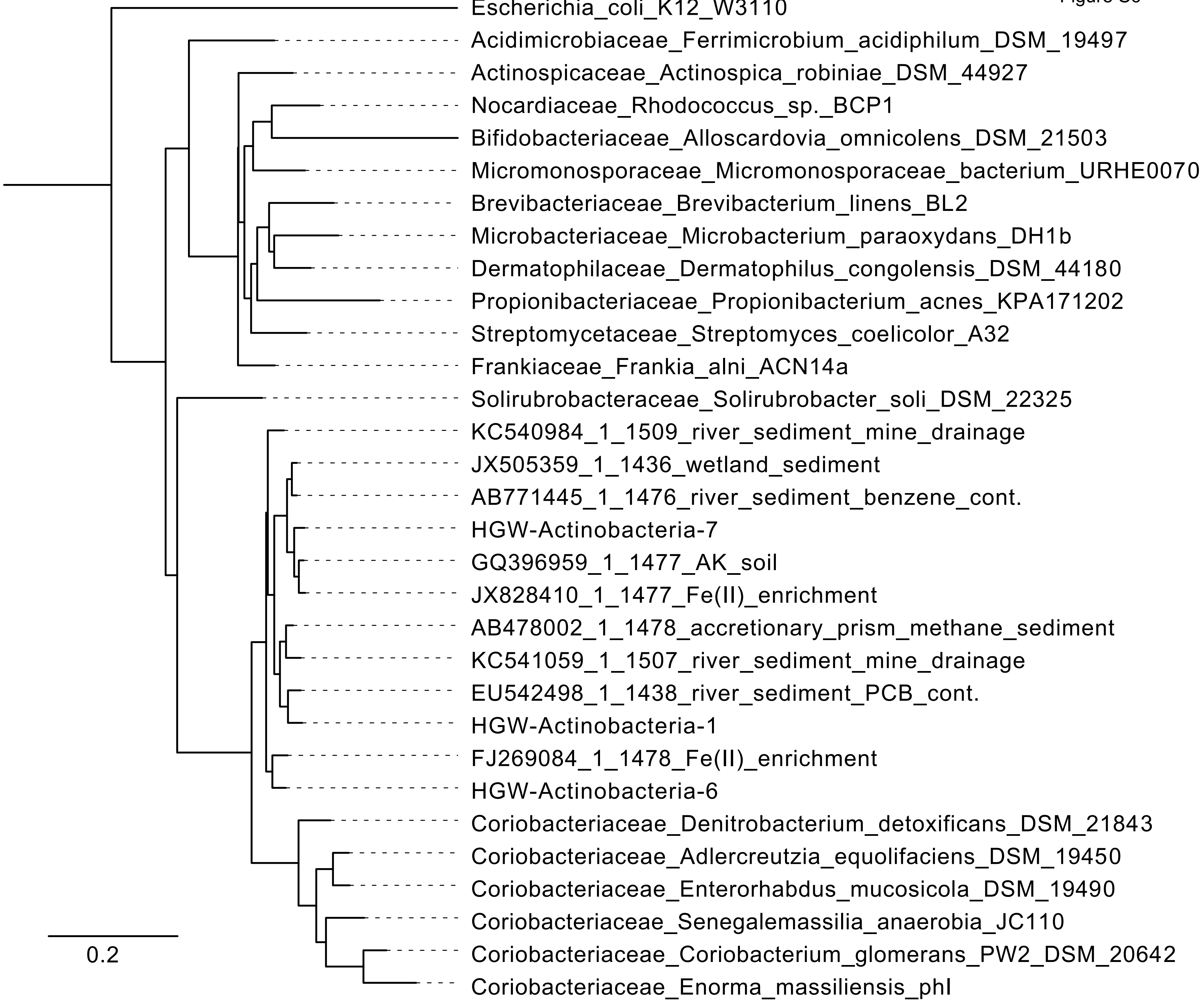

Supplement: Supplementary Table S1 [file ismej201739x2.pdf]
